# Circadian rhythm disruption aggravates high-fat diet-induced NAFLD through gut microbiota alterations in mice

**DOI:** 10.3389/fmicb.2026.1916861

**Published:** 2026-09-09

**Authors:** Mengxia Sun, Jia Dong, Mingli Su, Huiwei Liu, Qian Sun, Keyi Lin, Run Yu, Hui Yang, Jingjing Lin, Jian Chen, Jianwei Shen, Hua Ye

**Affiliations:** 1 The Affiliated Lihuili Hospital of Ningbo University, Ningbo, China; 2 Cixi People's Hospital, Wenzhou Medical University, Ningbo, China; 3 Women and Children's Hospital of Ningbo University, Ningbo, China; 4 The 72nd Army Hospital of the People's Liberation Army of China, Huzhou, China; 5 Health Science Center, Ningbo University, Ningbo, China; 6 Xiangshan Red Cross Taiwan Compatrion Hospital Medical and Health Group, Ningbo, China

**Keywords:** circadian rhythm disruption, dysbiosis, fecal microbiota transplantation, gut microbiota, gut–liver axis, high-fat diet, metabolic disorders, non-alcoholic fatty liver disease

## Abstract

**Background:**

Non-alcoholic fatty liver disease (NAFLD) is closely associated with gut microbiota dysbiosis, and circadian rhythm disruption (CRD) has been reported to aggravate metabolic disorders. However, the contribution of gut microbiota alterations to CRD-associated deterioration of NAFLD remains incompletely understood.

**Objective:**

This study aimed to investigate the effects of CRD on gut microbiota alterations and hepatic metabolic abnormalities in high-fat diet (HFD)-induced NAFLD mice, and to explore whether microbiota modulation could influence CRD-associated disease progression.

**Methods:**

Male C57BL/6J mice were divided into three groups: normal diet with normal light-dark cycle (ND-LD), HFD with normal light-dark cycle (HFD-LD), and HFD with disrupted light-dark cycle (HFD-CRD). Food intake, locomotor activity, and body weight were monitored. After 14 weeks, samples were collected following euthanasia. The remaining HFD-LD mice were further divided into two groups: fecal microbiota donor and normal light-dark cycle with saline gavage (LD-NS). The remaining HFD-CRD mice were assigned to either disrupted light-dark cycle with saline gavage (CRD-NS) or disrupted light-dark cycle with fecal microbiota transplantation (CRD-FMT). After another 14 weeks, samples were again collected following euthanasia. Serum biochemical parameters, liver histology, inflammatory markers, and gut microbiota composition based on 16S rRNA sequencing were analyzed.

**Results:**

HFD induced NAFLD, while CRD exacerbated metabolic dysfunction, liver injury, and gut microbiota alterations. Over time, the impact of CRD on glucose metabolism and liver injury diminished. CRD further altered gut microbiota in NAFLD mice, reducing microbial diversity and increasing pro-inflammatory taxa. FMT from HFD-LD donors partially reshaped gut microbiota composition of CRD mice and attenuated several CRD-associated metabolic and hepatic abnormalities, although its effects on body weight and glucose metabolism were limited.

**Conclusion:**

These findings suggest that gut microbiota alterations contribute to CRD-associated aggravation of HFD-induced NAFLD, although further studies are required to identify specific microbial mediators.

## 1 Introduction

Non-alcoholic fatty liver disease (NAFLD) is a prevalent chronic liver disorder closely associated with metabolic syndrome, obesity, and dyslipidemia (Milic et al., 2014). High-fat diet (HFD) is a well-established factor contributing to the onset and progression of NAFLD by promoting lipid accumulation, inflammation, and insulin resistance (Hydes et al., 2021). Emerging evidence suggests that the gut microbiota plays a crucial role in NAFLD pathogenesis through its influence on lipid metabolism, bile acid composition, and systemic inflammation (Chen and Vitetta, 2020). Disruptions in the gut microbiota, known as gut dysbiosis, have been implicated in the exacerbation of NAFLD (Zhao et al., 2024).

CRD is another critical but often overlooked factor contributing to metabolic disorders, including NAFLD. Shift work, irregular sleep patterns, sleep deprivation, altered light exposure, jet lag, and other lifestyle disruptions associated with modern society can disturb the body's internal clock, leading to dysregulated metabolism, increased risk of metabolic disorders, and even cancer (Kim et al., 2018; Bae et al., 2019; Ye et al., 2021). Previous studies have demonstrated that CRD aggravates lipid metabolism disorders and promotes liver inflammation (Jouffe et al., 2022), yet its impact on gut microbiota in NAFLD remains unclear. We found that rhythm restoration didn't improve metabolism or steatosis in HFD-fed mice (Zhao et al., 2024). Given the intricate crosstalk between the circadian system and gut microbiota (Matenchuk et al., 2020), understanding their interaction in NAFLD pathogenesis is essential for identifying novel therapeutic strategies.

Dysregulated gut microbiota can promote the progression of NAFLD through several mechanisms. Previous studies have suggested that microbiota-derived metabolites, including lipopolysaccharides (LPS) and short-chain fatty acids (SCFAs), may participate in gut–liver axis regulation through pathways such as TLR4/NF-κB signaling; however, these mechanisms were not directly assessed in the present study (Grant et al., 1991; Kang et al., 2023; Ding et al., 2019). Multiple studies have shown that both HFD and CRD can lead to gut microbiota dysbiosis (Ding et al., 2022). It was demonstrated that chronic jet lag exacerbates alterations in gut microbiota composition, structure, and 24-h oscillatory rhythms in mice fed a high-fat, high-sugar diet, indicating that CRD can aggravate HFD-induced gut microbiota imbalance (Zheng et al., 2023). Unlike phase-shift jet lag models, our protocol used repeated alterations between light-dark exposure and continuous darkness. This paradigm was selected to induce environmental CRD but may not fully reproduce human shift-work conditions. Therefore, comparisons with jet lag models should be interpreted cautiously.

In this study, we investigated the effects of CRD on gut microbiota composition and NAFLD progression in HFD-fed mice. We hypothesized that CRD exacerbates NAFLD by inducing gut microbiota dysbiosis, thereby impairing lipid metabolism and promoting liver inflammation. Furthermore, we investigated whether FMT from HFD-fed mice maintained under normal circadian rhythm could modulate gut microbiota alterations and influence CRD-associated NAFLD phenotypes. Our findings provide further insights into the gut–liver axis and highlight the potential contribution of gut microbiota modulation to the interaction between CRD and metabolic disorders.

## 2 Materials and methods

### 2.1 Animal experimental design

This study was conducted in accordance with experimental animal regulations and received ethical approval from Ningbo University. The first phase of the study is the establishment of the CRD-HFD NAFLD mouse model. After dietary and environmental adaptation, male C57BL/6J mice were randomly assigned to three groups. ND-LD group (*n* = 6): normal chow with a standard 12-h light/12-h dark cycle. HFD-LD group (*n* = 17): high-fat diet (60% fat, Research Diets, USA, formula D12492) with a standard 12-h light/12-h dark cycle. HFD-CRD group (*n* = 18): HFD with a disrupted circadian rhythm (12-h light/12-h dark for 3 days, followed by 24-h dark for 4 days). The mice were housed for 14 weeks with *ad libitum* access to food and water. Food intake, water intake, resting activity (5 min), and body weight (BW) were monitored weekly during light cycle shifts. After 14 weeks, fresh fecal samples of six mice from each group (all ND-LD, randomly selected HFD-LD, and HFD-CRD) were collected and stored at −80 °C. These mice were euthanized the next day at Zeitgeber Time 0 (ZT0, 8:00 AM). Serum, liver, and epididymal fat samples were collected. Half of the largest liver lobe was fixed in formalin, and the remaining tissues were stored at −80 °C. Behavioral monitoring was conducted during Phase 1 to verify the establishment of the CRD model. These parameters were not assessed during phase 2.

**Figure 1:**
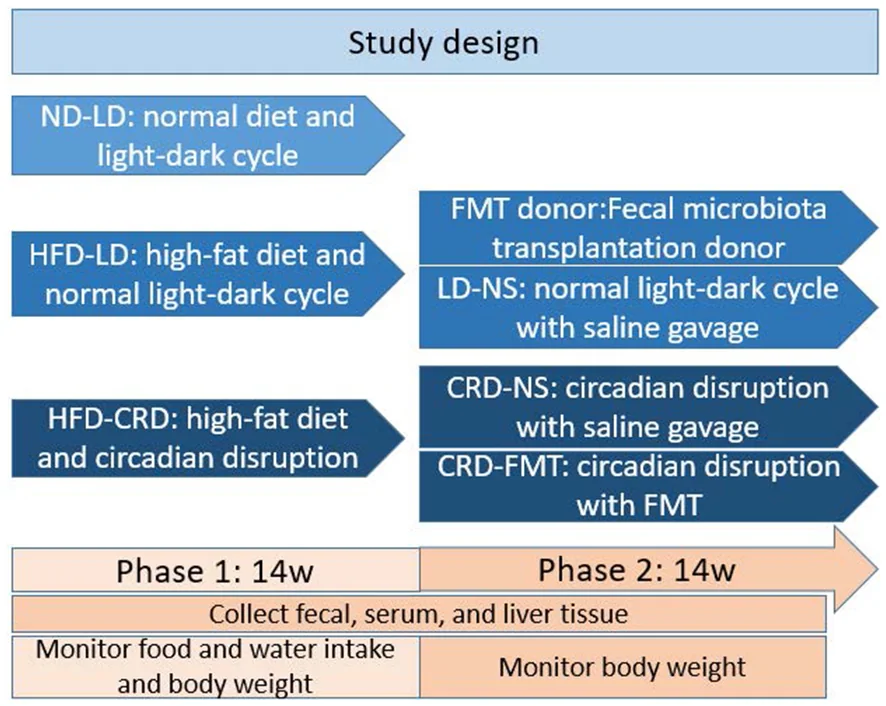
Schematic diagram of the two-phase experimental design.

The second phase of the study is investigating the effect of FMT from HFD-LD mice on NAFLD progression in HFD-CRD mice. The remaining HFD-LD mice (*n* = 11) were maintained on an HFD under a normal light-dark cycle and randomly divided into two groups. The FMT donor group (*n* = 5) served as the source of fecal microbiota. LD-NS group (*n* = 6) received normal saline gavage twice weekly. The remaining HFD-CRD mice were randomly assigned to two groups. CRD-NS group (*n* = 6) received normal saline gavage twice weekly. CRD-FMT group (*n* = 6) received FMT twice weekly via oral gavage using feces from the FMT donor group. We previously published a standardized method for gut microbiota extraction and isolation (Mi et al., 2023). In this study, we applied this method to ensure the reproducibility of microbiota processing. These three groups received gavage at the time point of light cycle shifts. BW was measured weekly during light cycle shifts. After 14 weeks, all mice were euthanized, and fecal, serum, and liver samples were collected using the same procedures as in the first phase. The study design is shown in Figure 1.

We acknowledge that a heat-inactivated fecal slurry control was not included in the present study. Therefore, the observed effects of FMT cannot be exclusively attributed to viable microorganisms, as other fecal components may contribute to the phenotype.

### 2.2 Monitoring general mouse health status

During the first phase, weekly monitoring of food intake, water intake, BW, and resting activity (5 min) was performed whenever the light cycle was changed. Each cage contained 3 mice, and data from cages with fewer than 3 mice were excluded. In the second phase, BW was measured regularly.

### 2.3 Measurement of serum indicators

Serum levels of alanine aminotransferase (ALT), aspartate aminotransferase (AST), total cholesterol (TC), triglycerides (TG), high-density lipoprotein cholesterol (HDL-c), low-density lipoprotein cholesterol (LDL-c), and glucose (GLU) were measured using the Multiskan GO full-wavelength microplate reader (Thermo Fisher Scientific, United States). The measurement procedure followed the instructions in the reagent kit from Ningbo Meikang Biotechnology Co., Ltd.

### 2.4 Liver tissue histopathological analysis

Liver tissues fixed in formalin were dehydrated in a gradient concentration of alcohol and xylene, embedded in paraffin, sectioned, and stained with hematoxylin and eosin (HE). For histological evaluation, one representative liver section was prepared from the same anatomical location of the left hepatic lobe from each mouse. Histopathological examination was conducted using an Olympus BX51 optical microscope (LEICA, Wetzlar, GER). The histological evaluation was performed by an independent investigator blinded to the experimental groups. All animals included in the histological analysis were evaluated, with six mice analyzed per group (*n* = 6/group), and each mouse was considered as an independent biological replicate. The non-alcoholic fatty liver activity score (NAS) was assessed based on HE staining. The NAS was defined as the sum of the scores for steatosis (0–3), lobular inflammation (0–3), and ballooning degeneration (0–2), with a total score range from 0 to 8.

### 2.5 Realtime quantitative PCR

The total RNA from mice hepatic tissues (20 mg) was extracted using TransZol Up Total RNA Extraction Kit (TransGen Biotech, Beijing, China). The reverse transcription system (20 μL) included the following items: 5 × TransScript^®^ All-in-One SuperMix for qPCR 4 μL, total RNA 1 μg, gDNA Remover 1 μL, and RNase-free Water *x* μL. The cDNA synthesized was stored at −80 °C and subjected to analysis within 7 days.

The primer sequences were extracted from https://pga.mgh.harvard.edu/primerbank. Each 20 μL PCR system contained total cDNA 1 μL, 2 × PerfectStartTM Green qPCR SuperMix 10 μL, forward primer 0.4 μL, reverse primer 0.4 μL, Universal Passive Reference Dye 0.4 μL, and RNase-free Water 7.8 μL. Amplification was performed using a reaction cycle at 94 °C for 30 s, 94 °C for 5 s, and then 60 °C for 34 s, 40 cycles. The fluorescence signal was detected at the end of each cycle. 18S rRNA was used as an internal control, and 2^−Δ*ΔCt*^ was used to calculate the expression of the target genes. Primers for *Rev-erb*α, *Bmal1, Per1, Per2, Ror*α, and *18S rRNA* were shown in Supplementary Table 1.

### 2.6 Gut microbiota analysis

The gut microbiota analysis was entrusted to Shanghai OE Biotech Co., Ltd. The procedure is as follows. Genomic DNA from samples was extracted using a kit, and its concentration was detected by agarose gel electrophoresis and NanoDrop 2000. Using the genomic DNA as a template, specific primers with barcodes were used to amplify the sequencing region via PCR with Tks Gflex DNA polymerase (Takara). The target region for bacterial diversity identification was the 16S V3-V4 region (343F primer and 798R primer). PCR products were detected by electrophoresis, purified using magnetic beads, and used as templates for secondary PCR amplification. The products were again detected, purified, and the Qubit concentration of the PCR products was measured. Based on the concentration of the PCR products, equal amounts were pooled together for sequencing. Raw data were stored in FASTQ format. After sequencing, raw data sequences were processed using the Cutadapt software to trim primer sequences. The processed sequences were then subjected to quality filtering, denoising, merging, and chimera removal using the DADA2 pipeline in QIIME2. Representative sequences and ASV (Amplicon Sequence Variant) abundance tables were generated. The representative sequences of each ASV were selected and aligned with a database for annotation. The 16S rRNA gene sequencing data were compared with the Silva (version 138) database. LEfSe analysis was used for biomarker discovery, while differential abundance significance was interpreted based on Benjamini–Hochberg FDR-adjusted results.

### 2.7 Statistical analysis

The experimental results are presented as mean ± standard deviation (mean ± SD). Statistical analysis was conducted using SPSS 25.0 (IBM Corp., Chicago, IL, USA) software, and figures were generated using GraphPad Prism 9.4.1 (GraphPad Software, Boston, MA, USA). For NAS subscores comparisons, the Kruskal–Wallis test with Dunn's *post hoc* test with Bonferroni correction was applied. A *p*-value < 0.05 was considered statistically significant. Significance levels are indicated as follows. *p* > 0.05 (ns): no significant difference, *p* < 0.05 (^*^/#), *p* < 0.01 (^**^/##), *p* < 0.001 (^***^/###), *p* < 0.0001 (^****^/####). In the first phase: “^*^” indicates comparison with ND-LD, “#” indicates comparison with HFD-LD. In the second phase: “^*^” indicates comparison with LD-NS, “#” indicates comparison with CRD-NS.

## 3 Results

### 3.1 Behavioral and liver circadian clock changes in mice with CRD

**Figure 2:**
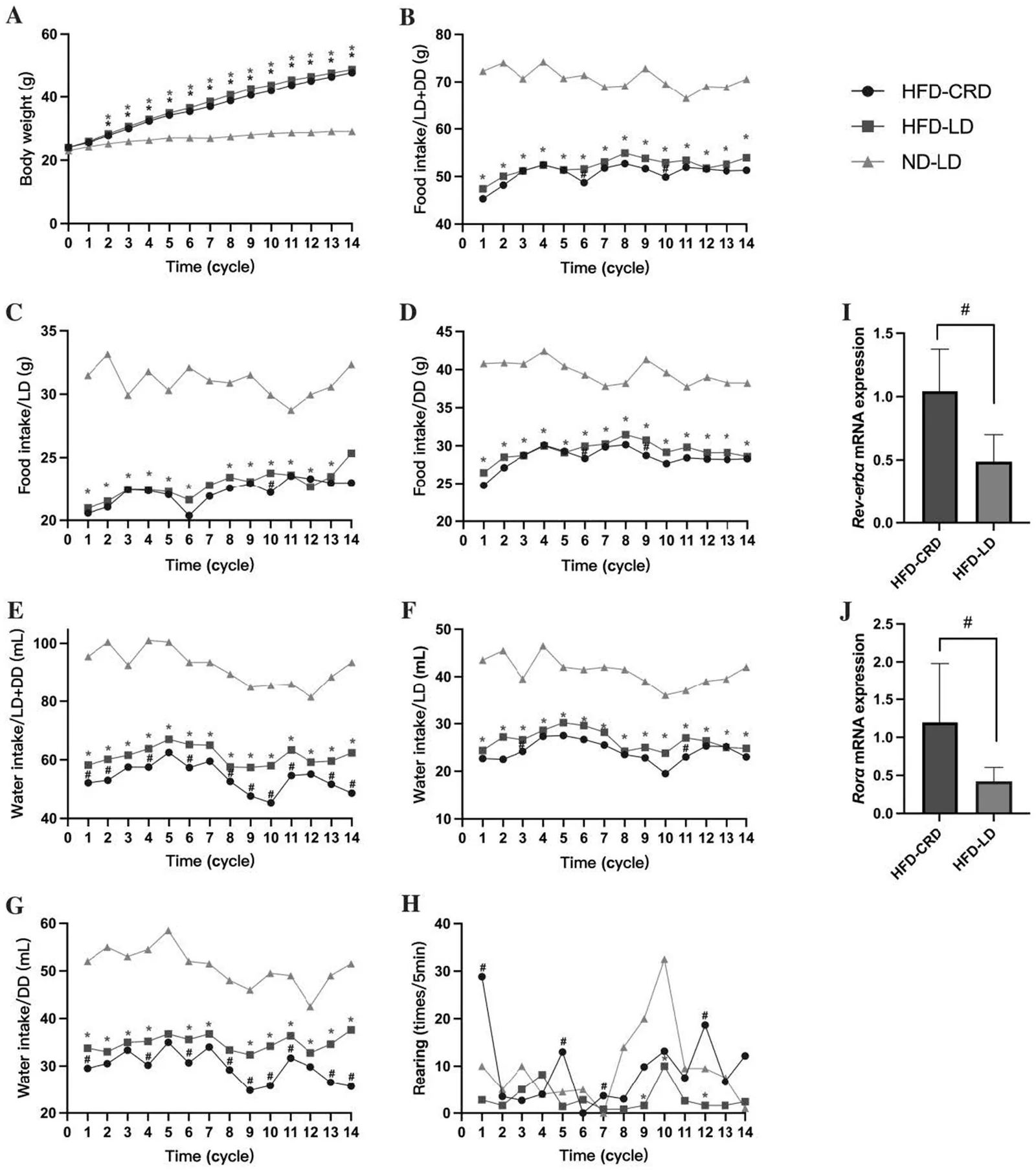
Circadian disruption exacerbates metabolic and behavioral changes in high-fat diet mice during the first phase. **(A)** Body weight. **(B)** Food intake at the end of each cycle. **(C)** Food intake per cycle under normal lighting conditions. **(D)** Food intake per cycle under abnormal lighting conditions. **(E)** Water intake per cycle. **(F)** Water intake under normal lighting conditions. **(G)** Water intake under abnormal lighting conditions. **(H)** Activity level of mice in a resting state within 5 min. **(I)** Liver mRNA expression of *Rev-erb*α. **(J)** Liver mRNA expression of *Ror*α. ^#^*P* < 0.05.

We observed the rapid weight gain in the HFD groups (Figure 2A). However, food intake was significantly lower in the HFD-LD group compared to the ND-LD group. After 14 weeks of CRD, the HFD-CRD group exhibited a significant reduction in food intake during the 6th and 10th cycles (weeks) compared to the HFD-LD group (Figure 2B). However, this pattern remained consistent under both normal lighting and total darkness conditions (Figures 2C, D), suggesting that food intake was minimally influenced by the lighting mode.

Water intake followed a similar trend, with significantly lower consumption in the HFD-LD group compared to the ND-LD group. Notably, the HFD-CRD group displayed a significant decrease in water intake across multiple time points, including the 1st, 2nd, 4th, 6th, 8th, 9th, 10th, 11th, 13th, and 14th cycles (Figure 2E). Unlike food intake, water intake was more sensitive to changes in the lighting environment, with total darkness further exacerbating the reduction (Figures 2F, G).

Locomotor activity was lower in the HFD-LD group compared to the ND-LD group. However, the HFD-CRD group exhibited significantly higher activity levels than the HFD-LD group during the 1st, 5th, 7th, and 12th cycles (Figure 2H).

At the molecular level, hepatic mRNA expression of *Rev-erb*α and *Ror*α was significantly elevated in the HFD-CRD group compared to the HFD-LD group (Figures 2I, J). However, the expression of *Bmal1, Per1*, and *Per2* showed numerical differences between the two groups, although these changes did not reach statistical significance (Supplementary Figure 1).

### 3.2 CRD exacerbates NAFLD in HFD mice

**Figure 3:**
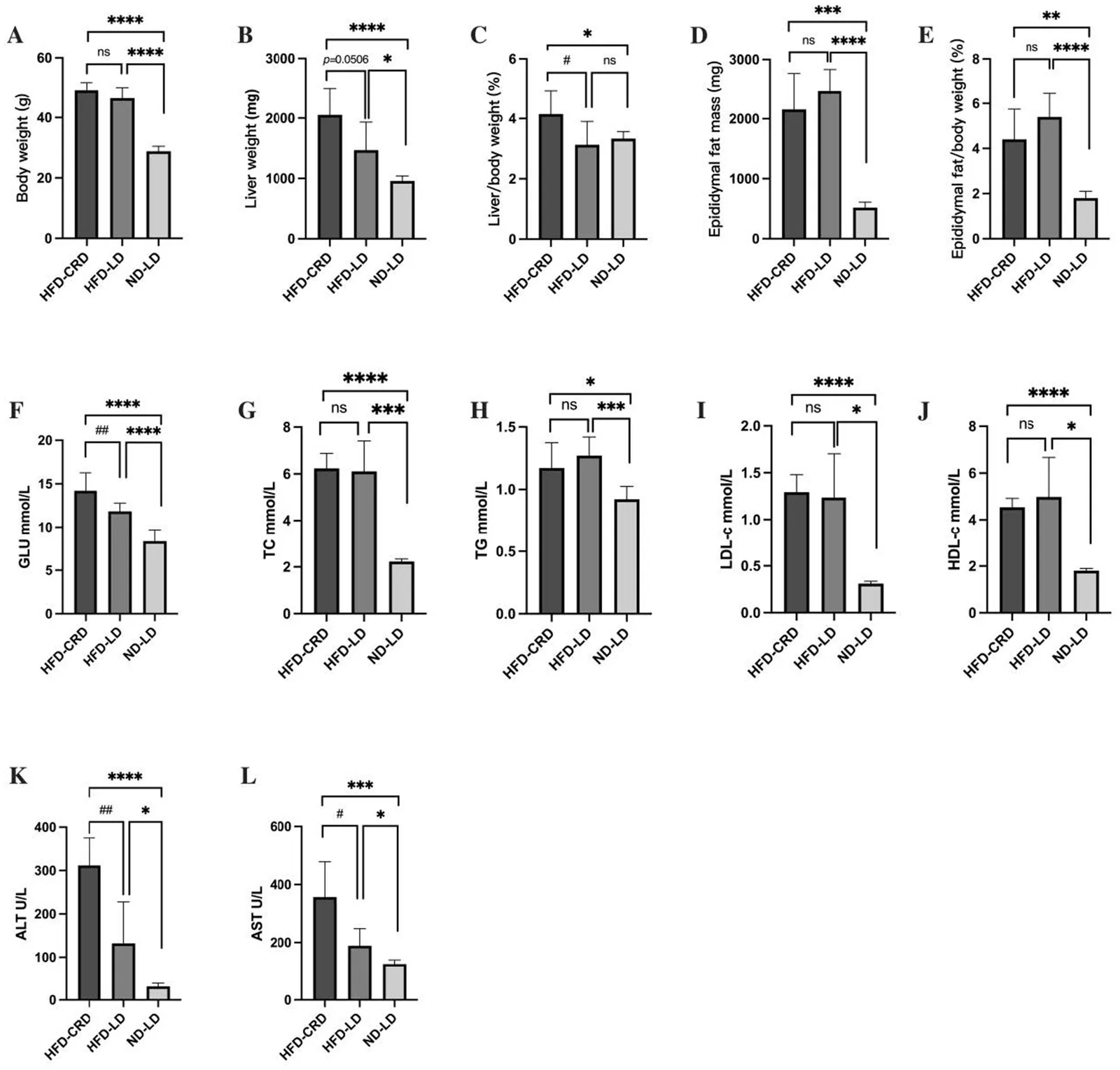
Effects of circadian rhythm disruption on metabolic and physiological parameters in mice during the first phase. **(A)** Body weight. **(B)** Liver weight. **(C)** Liver/body weight ratio. **(D)** Epididymal fat mass. **(E)** Epididymal fat/body weight ratio. **(F)** Serum glucose (GLU). **(G)** Serum total cholesterol (TC). **(H)** Serum triglycerides (TG). **(I)** Serum low-density lipoprotein cholesterol (LDL-c). **(J)** Serum high-density lipoprotein cholesterol (HDL-c). **(K)** Serum alanine aminotransferase (ALT). **(L)** Serum aspartate aminotransferase (AST). ^#^*P* < 0.05, ^*##*^*P* < 0.01. *P < 0.05, **P < 0.01, ***P < 0.001, ****P < 0.0001.

After 14 weeks, mice in the HFD-LD group exhibited significantly higher BW, liver weight (LW), epididymal fat weight (EFW), and EFW/BW ratio compared to those in the ND-LD group (Figures 3A–E). While the HFD-CRD group showed similar BW, EFW, and EFW/BW ratios to the HFD-LD group, their LW was notably higher. Additionally, the LW/BW ratio in the HFD-CRD group was significantly elevated compared to the HFD-LD group, suggesting that while HFD induces obesity, CRD further promotes liver enlargement in HFD mice.

The HFD-LD group displayed significantly increased serum glucose (GLU) levels and lipid profiles compared to the ND-LD group (Figure 3F). Notably, serum GLU levels were further elevated in the HFD-CRD group relative to the HFD-LD group, while their lipid profiles remained comparable (Figures 3G–J).

Serum ALT and AST levels were significantly elevated in the HFD-LD group compared to the ND-LD group (Figures 3K, L). Moreover, ALT levels in the HFD-CRD group were approximately 2.4-fold higher, and AST levels were approximately 2-fold higher compared to the HFD-LD group.

**Figure 4:**
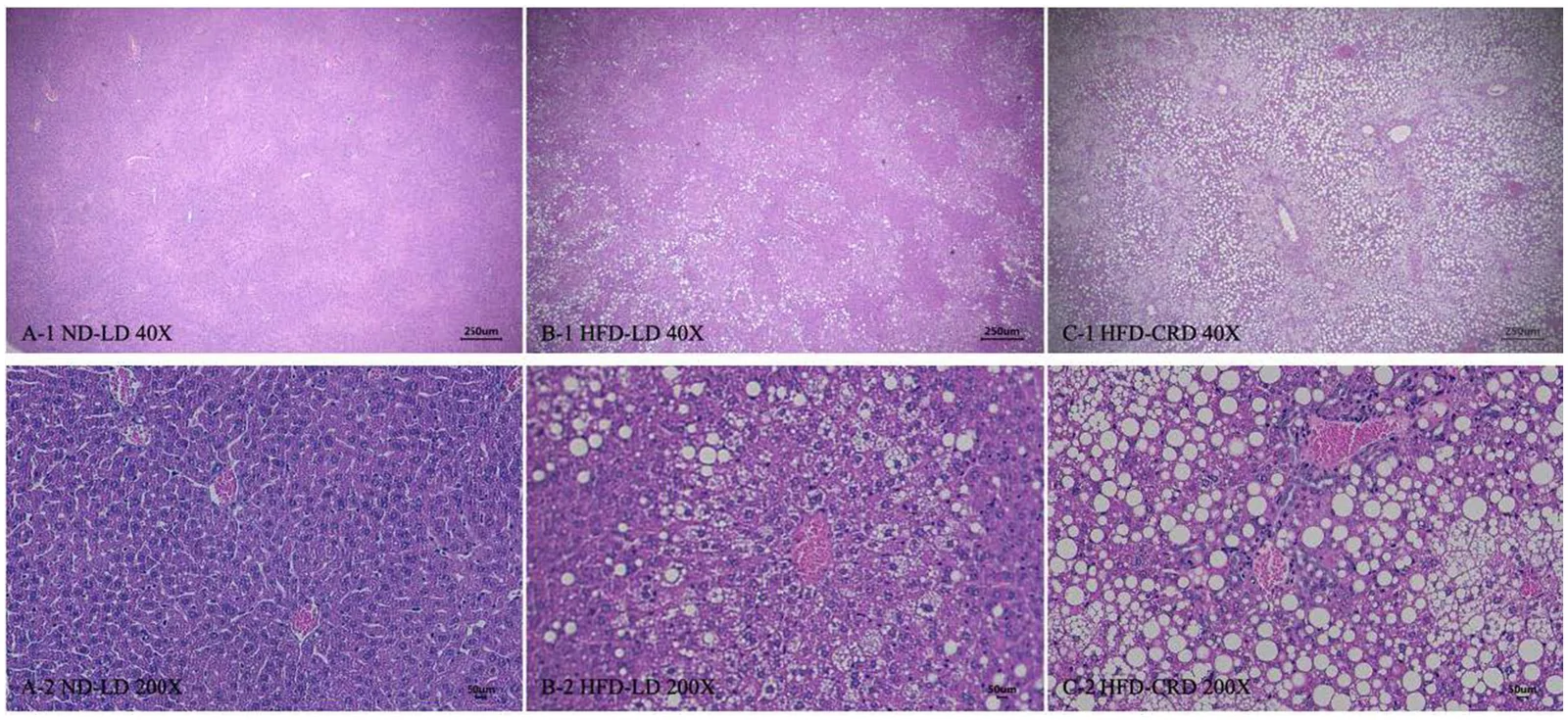
Mouse liver histopathology with HE staining in the first phase circadian rhythm disruption in **(A)** ND-LD group, **(B)** HFD-LD group, and **(C)** HFD-CRD group.

Histological analysis revealed that the ND-LD group exhibited intact liver lobule structures without signs of fat degeneration or inflammatory cell infiltration (Figure 4A). Based on NAS scores (Supplementary Table 2), the HFD-LD group displayed disrupted liver lobule organization, with fat degeneration primarily affecting hepatocytes in Zone III. However, no significant inflammatory cell infiltration, ballooning degeneration, necrotic foci, or fibrosis were observed (Figure 4B). In contrast, the HFD-CRD group showed more severe liver lobule disorganization, with fat accumulation extending into hepatocytes in Zone I (Figure 4C). Additionally, scattered necrotic foci and inflammatory cell infiltration were observed in the portal areas, although no significant ballooning degeneration or fibrosis was present.

### 3.3 CRD leads to gut microbiota dysbiosis in HFD-induced NAFLD mice

**Figure 5:**
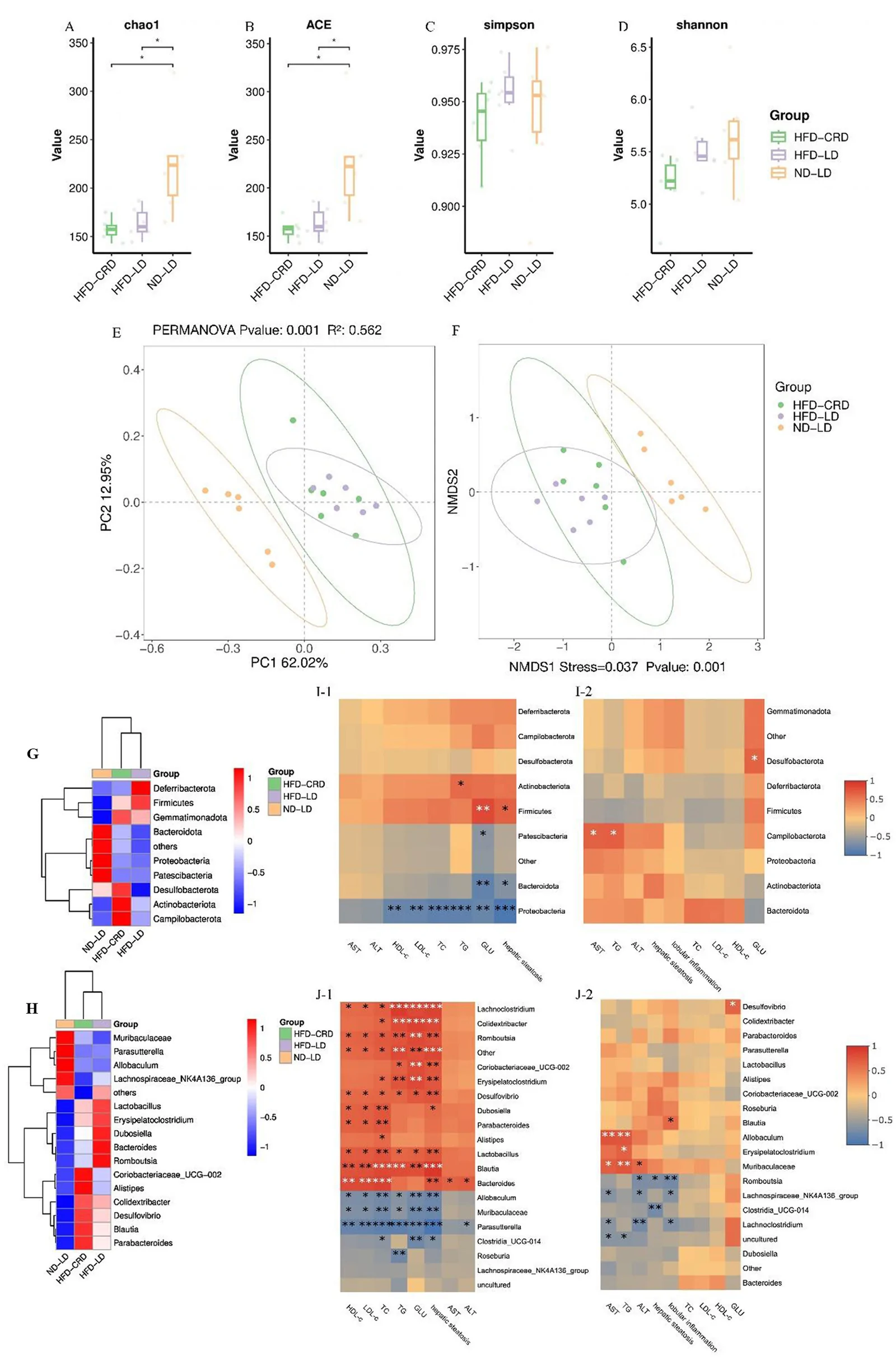
Effects of circadian rhythm disruption on gut microbial diversity and community structure in the first phase. **(A)** Chao1 index, **(B)** abundance-based coverage estimator (ACE) index, **(C)** Simpson index, **(D)** Shannon index, **(E)** principal coordinate analysis (PCoA), and **(F)** non-metric multidimensional scaling (NMDS) analysis show the differences between groups. **(G)** Phylum-level and **(H)** genus-level differential taxa abundance heatmap. Correlation analysis between **(I)** phylum-level and **(J)** genus-level microbial relative abundance and clinical indices: **(I-1, J-1)** HFD-LD vs. ND-LD groups; **(I-2, J-2)** HFD-CRD vs. HFD-LD groups. *P < 0.05, **P < 0.01, ***P < 0.001.

In the HFD-LD group, alpha diversity was significantly lower than in the ND-LD group, as indicated by a marked reduction in Chao1 and abundance-based coverage estimator (ACE) indices (Figures 5A,B). Alpha diversity was lower in the HFD-CRD group than in the HFD-LD group; however, no statistically significant difference was observed between the two groups (Figures 5C, D). Principal coordinate analysis (PCoA) and non-metric multidimensional scaling (NMDS) analyses revealed significant differences in microbial community composition between the groups (Figures 5E, F). Additionally, the composition of significantly enriched gut microbiota species varied among the three groups. CRD administration in HFD-induced NAFLD murine models demonstrated a marked increase in the relative abundance of *Actinobacteria, Desulfobacterota, Campylobacterota*, and *Blautia*, concurrent with reduced proportions of *Lachnospiraceae* NK4A136 *group, Acetatifactor, Odoribacter*, and *Romboutsia et al*. (Figures 5G, H). Additionally, several phylum- and genus-level taxa showed significant correlations with serum biochemical parameters and hepatic pathological features (Figures 5I, J).

### 3.4 Fecal microbiota transplantation from HFD-LD mice can reshape gut microbiota in HFD-CRD mice

**Figure 6:**
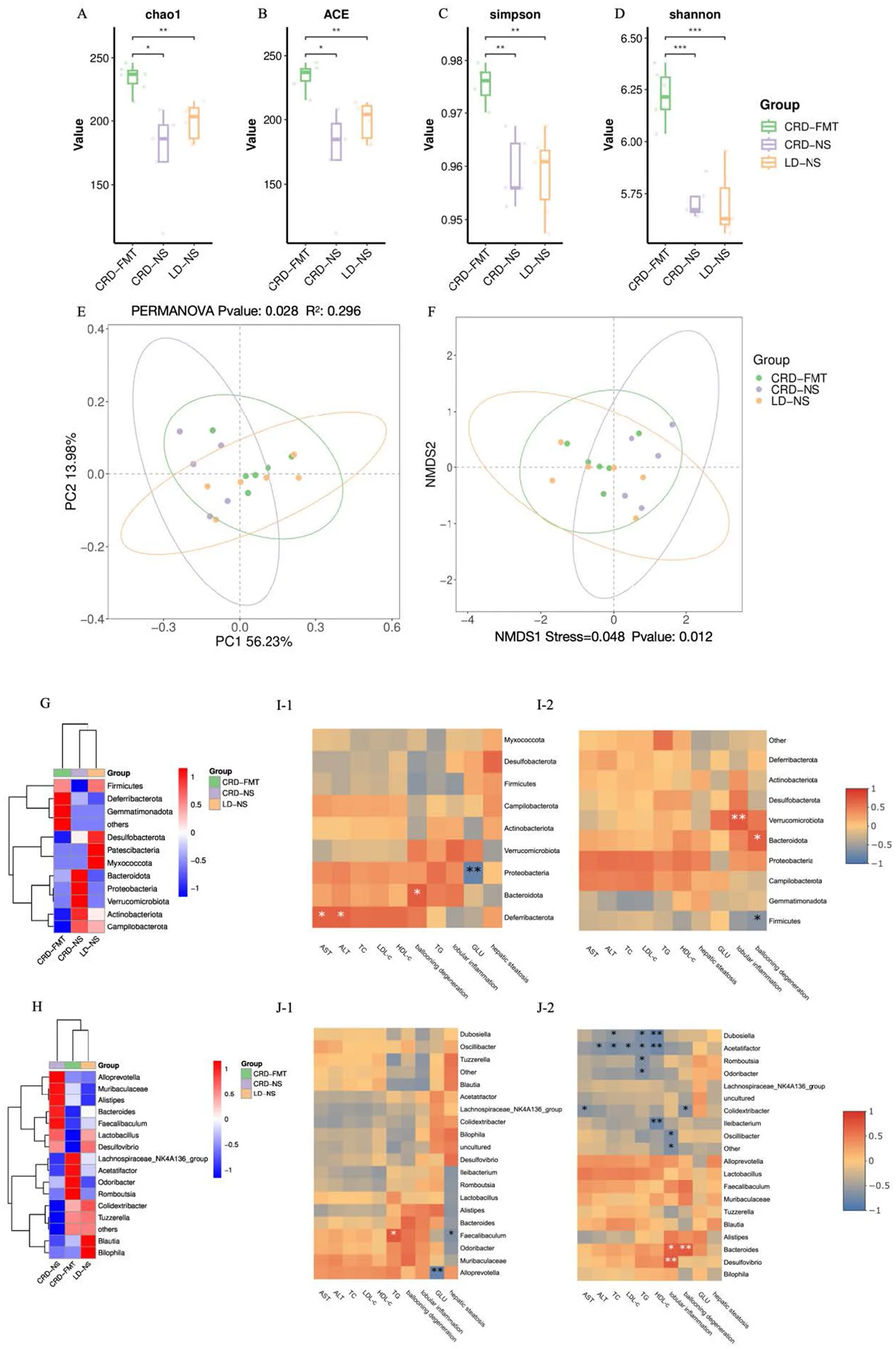
Effects of circadian rhythm disruption (CRD) on gut microbial diversity and community structure in the second phase. **(A)** Chao1 index, **(B)** abundance-based coverage estimator (ACE) index, **(C)** Simpson index, **(D)** Shannon index, **(E)** principal coordinate analysis (PCoA), and **(F)** non-metric multidimensional scaling (NMDS) analysis show the differences between groups. **(G)** Phylum-level and **(H)** genus-level differential taxa heatmap. Correlation analysis between **(I)** phylum-level and **(J)** genus-level microbial relative abundance and clinical indices: **(I-1, J-1)** CRD-NS vs. LD-NS groups; **(I-2, J-2)** CRD-FMT vs. CRD-NS groups.

After 14 weeks of FMT, alpha diversity in the CRD-NS group was lower than in the LD-NS group, though the difference was not statistically significant. However, the CRD-FMT group exhibited a significant increase in alpha diversity compared to both the CRD-NS and LD-NS groups (Figures 6A–D). PCoA and NMDS analyses revealed significant differences in microbial community diversity between groups. Notably, the CRD-NS and LD-NS groups formed distinct clusters, while the CRD-FMT group clustered at the intersection of the CRD-NS and LD-NS groups (Figures 6E, F).

Furthermore, correlations between microbiota relative abundance and metabolic indicators were analyzed. Compared with the CRD-NS group, mice receiving FMT from HFD-LD donors exhibited altered relative abundances of *Dubosiella, Acetatifactor, Romboutsia, Odoribacter*, and *Ileibacterium*, which were associated with changes in lipid metabolism-related parameters following FMT. Additionally, the relative abundances of *Firmicutes, Acetatifactor, Colidextribacter*, and *Oscillibacter* showed associations with liver injury-related indicators, suggesting that these taxa may be involved in CRD-associated metabolic alterations (Figures 6G–J).

### 3.5 FMT-associated microbiota modulation may influence CRD-associated NAFLD phenotypes

**Figure 7:**
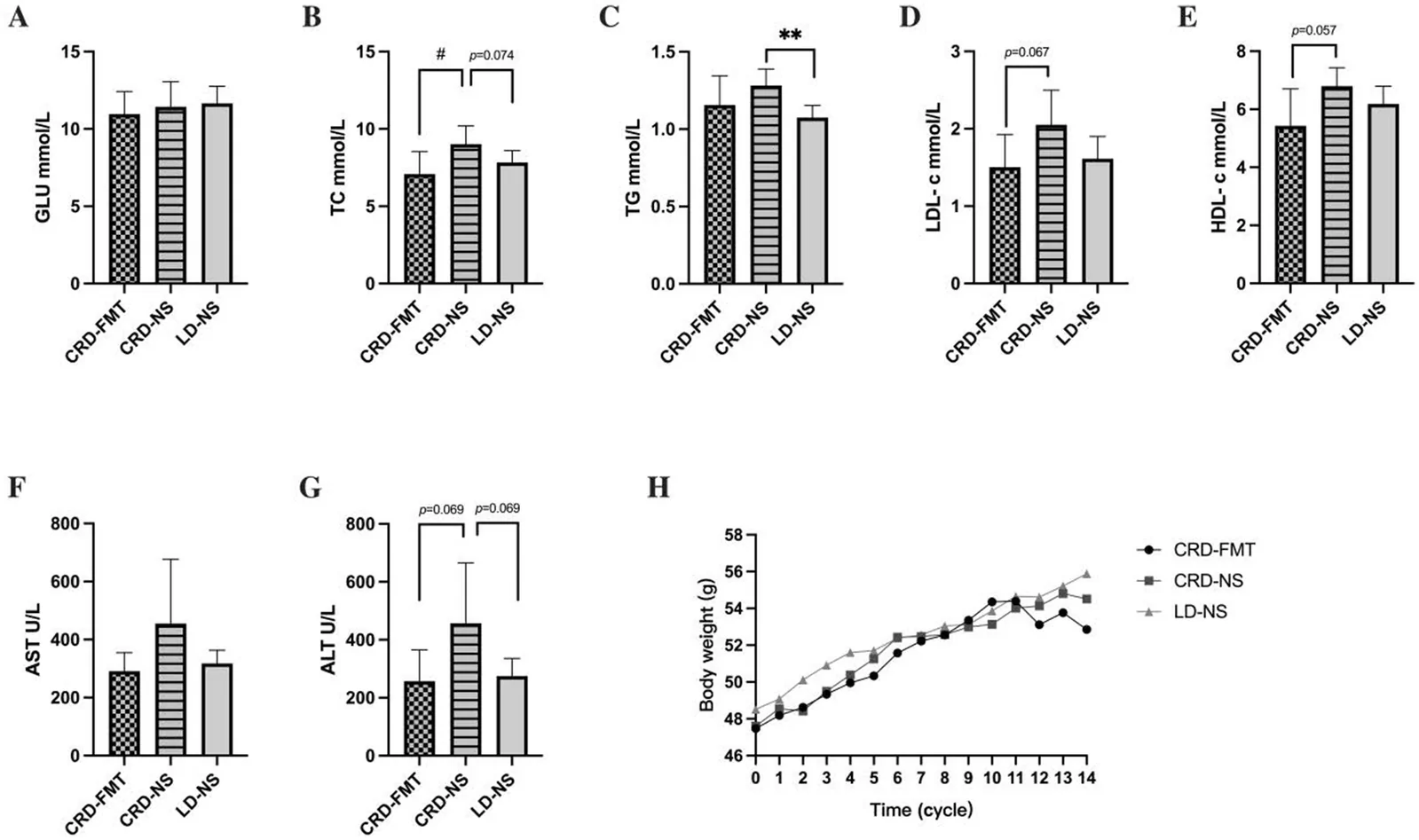
Effects of circadian rhythm disruption (CRD) and fecal microbiota transplantation (FMT) on metabolic parameters in mice. **(A)** Serum glucose (GLU). **(B)** Serum total cholesterol (TC). **(C)** Serum triglycerides (TG). **(D)** Serum low-density lipoprotein cholesterol (LDL-c). **(E)** Serum high-density lipoprotein cholesterol (HDL-c). **(F)** Serum alanine aminotransferase (ALT). **(G)** Serum aspartate aminotransferase (AST). **(H)** Body weight. ^#^*P* < 0.05. **P < 0.01.

Following FMT, GLU levels did not differ significantly among groups (Figure 7A). Serum lipid levels were reduced in the CRD-FMT group compared to the CRD-NS group, with a significant decrease in serum TC levels (Figure 7B). However, the CRD-NS group exhibited higher serum lipid levels than the LD-NS group, with a significant increase in serum TG levels and numerical increases in LDL-c and HDL-c with no significance (Figures 7C–E). This suggests that prolonged CRD had little impact on glucose homeostasis but exacerbated lipid metabolism disorders in HFD-NAFLD mice.

Serum ALT and AST levels showed numerical increases in the CRD-NS group compared with the LD-NS group, while the CRD-FMT group exhibited lower ALT and AST levels compared with the CRD-NS group; however, these differences did not reach statistical significance (Figures 7F, G). The CRD-NS group showed higher BW, LW, EFW, and EFW/BW ratios compared with the LD-NS group, while the CRD-FMT group showed lower values than the CRD-NS group. However, no statistically significant differences were observed among groups (Figure 7H).

**Figure 8:**
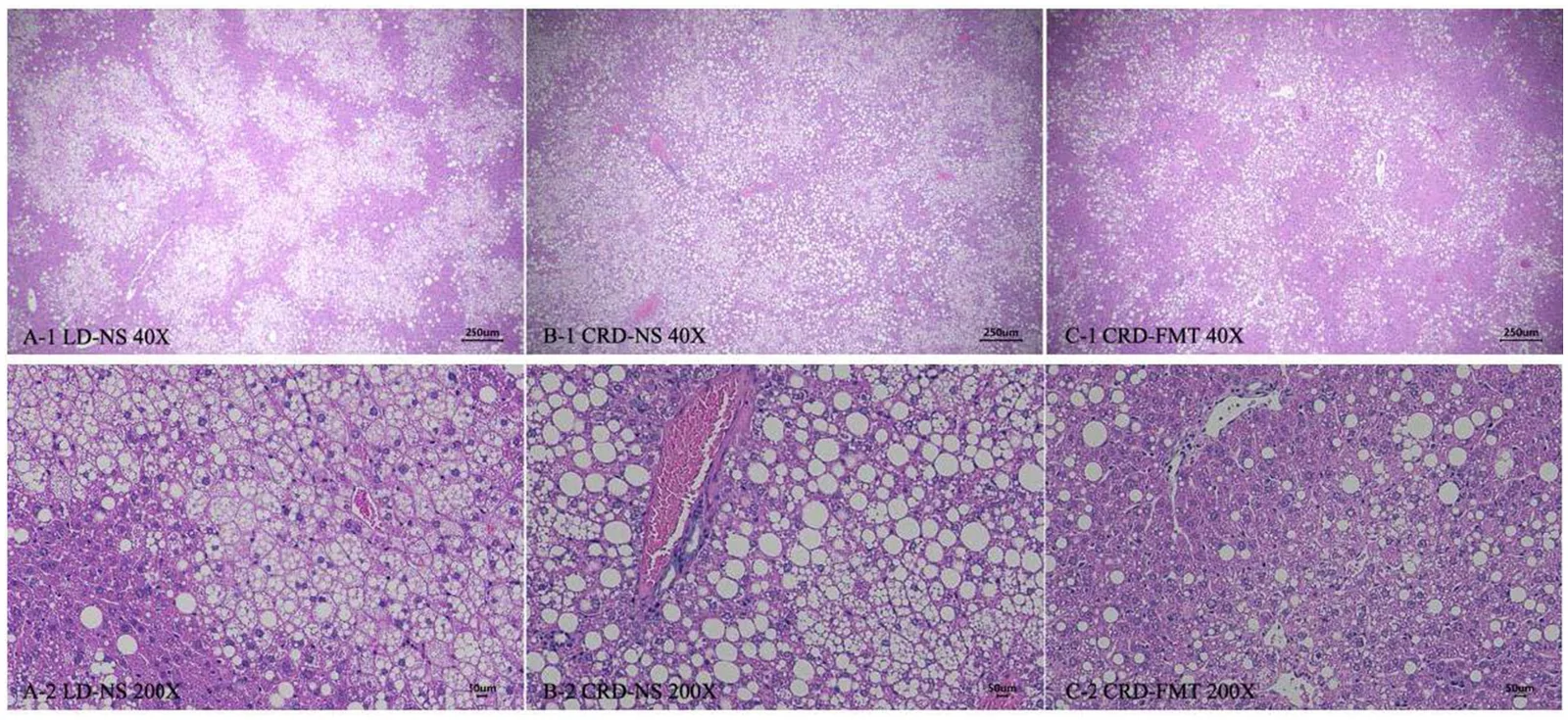
Mouse liver histopathology with HE staining in the second phase in **(A)** LD-NS group, **(B)** CRD-NS group, and **(C)** CRD-FMT group.

Liver histopathology and NAS assessments (Figure 8 and Supplementary Table 3) suggested that the LD-NS group exhibited disrupted liver lobule architecture, widespread hepatocyte steatosis, mild inflammatory cell infiltration, and hepatocyte ballooning, with no evidence of necrosis or fibrosis. Compared to the LD-NS group, the CRD-NS group displayed more severe liver lobule structural disorganization, similar levels of hepatocyte steatosis, significantly increased portal area inflammation, scattered necrotic foci in some lobules, and mild hepatocyte ballooning, but no significant fibrosis.

Compared with the CRD-NS group, the CRD-FMT group showed reduced hepatocyte steatosis scores (Figure 8). Although lobular inflammation remained higher in CRD-FMT mice compared with LD-NS mice, it was significantly decreased compared with the CRD-NS group. No apparent ballooning degeneration or fibrotic lesions were observed among the groups.

## 4 Discussion

In this study, we investigated the effects of CRD on NAFLD progression and explored whether FMT-associated modulation of gut microbiota composition could alleviate CRD-associated metabolic and hepatic abnormalities. Our findings suggest that CRD exacerbates hepatic lipid accumulation, inflammation, and gut microbiota dysbiosis. FMT partially reshapes microbial diversity and alleviates some metabolic disturbances associated with NAFLD and hepatic inflammation.

Light is the most important zeitgeber that regulates circadian rhythms. It influences circadian entrainment by promoting CREB phosphorylation in the suprachiasmatic nucleus (SCN) neurons through the retina, thereby inducing clock gene expression and altering the autonomous activity rhythms of mammals (Ginty et al., 1993). We found that CRD significantly reduced both food and water intake in HFD-fed mice compared to those with normal circadian rhythms. However, CRD had little impact on BW in HFD mice. These findings are consistent with the previous studies (Summa et al., 2013; Ferrell, 2023). Rev-erbα (NR1D1) and Rorα (RAR-related orphan receptor alpha) are key regulators of the circadian clock and metabolism (Gerhart-Hines and Lazar, 2015). The altered circadian cycle in HFD-fed NAFLD mice resulted in significant up-regulation of hepatic *Rev-erb*α and *Ror*α expression at ZT0 compared to the normal rhythm HFD group, indicating molecular-level circadian dysregulation. Notably, up to 50% of liver metabolites exhibit circadian rhythms that are coupled with Rev-erb expression (Feng et al., 2011). Rorα is known to play multiple roles in lipid metabolism, hepatic oxidative stress, and inflammation (Chauvet et al., 2011). However, *Bmal1, Per1*, and *Per2* gene expression at ZT0 showed no significant difference between the HFD-CRD and HFD-LD groups. This is different from a previous finding (Xie et al., 2017). Potential explanations include differences in experimental design, as they used a normal chow diet instead of an HFD. Furthermore, they performed clock gene expression measurements every 6 h throughout a 24-h cycle (Zheng et al., 2023), while our study only examined expression levels at ZT0, limiting our ability to assess the full oscillatory dynamics.

The influence of CRD extends beyond clock gene expression to metabolic regulation, including glucose and lipid metabolism. By the end of the first phase of our study, HFD-CRD mice exhibited significantly higher GLU levels than HFD-LD mice, whereas lipid metabolism showed no significant changes. These results are similar to a previous report (Zheng et al., 2023). Interestingly, the EFW and the EFW/BW ratio showed numerical decreases in HFD-CRD compared with the HFD-LD group, although these differences were not statistically significant. This may be attributed to reduced food and water intake and increased physical activity. By the end of the second phase, GLU levels in CRD-NS mice had no significant change compared to LD-NS mice, but lipid metabolic disorders worsened, as evidenced by a significant increase in TG levels.

Biological clocks play a critical role in regulating inflammatory responses through time-gated mechanisms (Carter et al., 2016). It was found that prolonged sleep deprivation induces up-regulation of pro-inflammatory cytokines and chemokines, triggering cytokine storm syndrome, which can lead to multi-organ failure and even death (Sang et al., 2023; Möller-Levet et al., 2013; Yan et al., 2024). Our study supports this conclusion at the liver level, as we found that prolonged CRD exacerbates liver injury in NAFLD mice. By the end of the second phase, CRD-NS mice exhibited significantly increased intrahepatic inflammatory cell infiltration compared to LD-NS mice. However, serum ALT and AST levels showed only a not significant increase. Potential explanations include: (1) both groups already exhibited extremely high baseline liver enzyme levels, making further increases less pronounced; (2) previous findings suggest that while ALT levels increase significantly after 8 weeks of CRD in HFD-fed mice, they do not show a significant difference after 16 weeks of CRD combined with a high-fat, high-fructose diet (Zheng et al., 2023; Christie et al., 2018). This suggests that although CRD continuously promotes NAFLD progression, its impact may diminish over time due to compensatory mechanisms, which warrant further investigation.

Moreover, we found that CRD significantly altered gut microbiota composition, resulting in reduced microbial diversity and distinct community structures in HFD-fed mice. By the end of the first phase, CRD exposure was associated with changes in gut microbiota composition, including increased relative abundances of *Actinobacteria, Desulfobacterota*, and *Campylobacterota*. Previous studies have reported that alterations in *Campylobacterota* abundance are associated with intestinal inflammation and chronic liver diseases, although the functional role of these taxa in CRD-associated NAFLD requires further investigation (Yan et al., 2023). Similarly, *Desulfobacterota* has been implicated in metabolic disorders and gut–liver axis regulation in previous studies (Matsumoto et al., 2026); however, whether these bacteria directly affect intestinal barrier function, LPS translocation, or hepatic TLR4/NF-κB signaling was not evaluated in the present study.

At the genus level, CRD was associated with increased abundances of *Blautia* and *Desulfovibrio*, accompanied by decreased abundances of taxa including the *Lachnospiraceae* NK4A136 group, *Acetatifactor, Odoribacter*, and *Romboutsia*. *Blautia*. Previous studies have linked alterations in *Blautia* abundance with metabolic disorders, whereas members of the *Lachnospiraceae* family have been reported to participate in short-chain fatty acid production and host metabolic regulation (Wang et al., 2021; Fitzgerald et al., 2025). However, the functional metabolites produced by these taxa were not directly measured in the current study, and their specific contributions to NAFLD progression remain to be determined.

By the end of the second phase, CRD-associated microbiota alterations were further evident, with significant beta-diversity shifts and distinct microbial community patterns between LD-NS and CRD-NS mice. Consistent with previous reports, CRD has been suggested to influence microbial community structure by affecting inflammatory-associated taxa and bacteria involved in metabolic regulation and gut barrier maintenance (Reynolds et al., 2017). Together, our findings indicate that CRD is associated with gut microbiota dysregulation during HFD-induced NAFLD progression. However, whether CRD exerts a stronger influence than HFD on microbiota alterations and disease progression requires further investigation using experimental designs specifically comparing these factors.

Given the association between microbial dysbiosis and metabolic disorders, FMT has been explored as a potential strategy for modulating gut microbial communities and improving metabolic phenotypes (Napolitano and Covasa, 2020). In our study, FMT from HFD-LD donors altered microbial community characteristics in CRD-FMT mice, including increased alpha diversity and changes in the relative abundance of several bacterial taxa. These microbiota alterations were accompanied by partial improvements in hepatic phenotypes, including reduced serum TC levels and attenuated hepatic inflammatory responses. However, BW and GLU levels remained largely unchanged, suggesting that FMT-associated microbiota modulation may not be sufficient to fully reverse all metabolic abnormalities induced by CRD in HFD-fed mice.

The interaction between circadian rhythm, gut microbiota, and NAFLD is complex. Previous studies have suggested that CRD may influence microbial community structure, intestinal barrier function, and microbial-derived inflammatory signals, thereby contributing to metabolic dysfunction (Aron-Wisnewsky et al., 2020). Similarly, gut microbiota alterations have been proposed to affect host metabolism through pathways involving LPS signaling, bile acid metabolism, and SCFA-related regulation (Olotu and Ferrell, 2024; Cui et al., 2025). We found the extent of improvement was limited, indicating that gut microbiota reshape alone may not fully counteract the metabolic consequences of CRD. However, these mechanisms were not directly evaluated in the present study. Therefore, the improvements observed following FMT should be interpreted as associations with microbiota modulation rather than direct evidence of microbiota-mediated regulation of these pathways.

Several limitations of the present study should be acknowledged. First, although FMT from HFD-LD donors partially attenuated several CRD-associated metabolic and hepatic abnormalities, HFD-LD mice themselves exhibited HFD-induced metabolic dysfunction and gut microbiota alterations. Therefore, the donor microbiota should not be considered a healthy microbial community, and the observed effects should be interpreted as modulation of CRD-associated microbial alterations rather than restoration of normal microbiota homeostasis. In addition, because a heat-inactivated fecal slurry control was not included, the specific contribution of viable donor microorganisms vs. non-microbial components of fecal material, such as microbial metabolites or host-derived factors, cannot be completely distinguished. Future studies incorporating heat-inactivated FMT controls, defined microbial consortia, or selective microbial depletion approaches are required to further clarify the causal role of specific microorganisms.

Second, although saline gavage was included as a procedural control during the FMT intervention phase, repeated oral gavage was not performed during phase 1. Therefore, the potential influence of gavage-associated stress on metabolic parameters during phase 2 and on comparisons across experimental phases cannot be completely excluded. Behavioral parameters, including food intake, water intake, and locomotor activity, were not monitored during the FMT intervention phase. Therefore, whether CRD-associated behavioral disruption persisted throughout phase 2 and whether FMT affected these circadian-related behaviors remain unclear. In addition, fecal microbiota profiling was performed at a single time point in each experimental phase. Therefore, although previous studies have demonstrated circadian oscillations of gut microbiota composition (Aron-Wisnewsky et al., 2020), the present study did not directly evaluate microbial rhythmicity or loss of diurnal oscillation. Longitudinal sampling across multiple Zeitgeber time points will be necessary to determine how CRD influences the temporal dynamics of gut microbial communities.

Third, the 16S rRNA sequencing approach used in this study identifies associations between microbial alterations and disease phenotypes but does not establish causal relationships for individual bacterial taxa. Although several differential taxa identified in our study have been implicated in metabolic regulation in previous reports, their specific functional contributions require further validation using approaches such as bacterial isolation, targeted colonization, metabolite supplementation, or microbiota depletion experiments. Moreover, potential mechanisms involving microbial-derived metabolites, including SCFAs and bile acids, as well as gut barrier integrity and inflammatory signaling pathways, were not directly measured in the current study and should be investigated in future work.

Finally, no formal power calculation was performed before the study, and the relatively small sample sizes, particularly in the FMT intervention and microbiota analyses, may limit the statistical power to detect subtle biological differences. Therefore, some findings should be interpreted as exploratory and require confirmation in larger independent cohorts. Furthermore, formal microbial source tracking or quantitative engraftment analysis was not performed, and therefore the extent to which donor-derived microorganisms contributed to recipient microbiota remodeling remains to be determined. Future studies with larger cohorts, multi-omics approaches, and comprehensive microbiota functional analyses will help further elucidate the mechanisms linking CRD, gut microbiota alterations, and NAFLD progression.

In conclusion, our study demonstrates that CRD exacerbates HFD-induced NAFLD and is accompanied by distinct alterations in gut microbiota composition. FMT from HFD-LD donors partially modified the microbial community structure and attenuated several CRD-associated hepatic abnormalities, including lipid metabolism disorders and inflammation. These findings highlight the gut microbiota as a potential mediator of circadian-metabolic interactions. However, given the limitations of the current experimental design, further studies are required to identify specific microbial mediators and mechanisms underlying the gut microbiota–circadian–liver axis, which may provide insights into potential strategies for managing CRD-associated metabolic disorders.

## Data Availability

The 16S rRNA sequencing data used in this study are available in the NCBI Sequence Read Archive (SRA) under the accession number PRJNA1344756. The data can be accessed at https://www.ncbi.nlm.nih.gov/bioproject/PRJNA1344756.
